# Dynamic Synergy Network Analysis Reveals Stage-Specific Regional Dysfunction in Alzheimer’s Disease

**DOI:** 10.3390/brainsci15060636

**Published:** 2025-06-12

**Authors:** Xiaoyan Zhang, Chao Han, Jingbo Xia, Lingli Deng, Jiyang Dong

**Affiliations:** 1 School of Information Science & Technology, Xiamen University Tan Kah Kee College, Zhangzhou 363105, China; xyzhang@xujc.com (X.Z.);; 2 School of Electronic Science and Engineering, National Model Microelectronics College, Xiamen University, Xiamen 361100, China; 33320221150321@stu.xmu.edu.cn; 3 Department of Information Engineering, East China University of Technology, Nanchang 330013, China

**Keywords:** resting-state fMRI, functional connectivity, Alzheimer’s disease, dynamic synergy network, graph theory

## Abstract

**Background:** Alzheimer’s disease (AD) is a prevalent neurodegenerative disorder characterized by progressive neurodegeneration and connectivity deterioration. While resting-state functional magnetic resonance imaging (fMRI) provides critical insights into brain network abnormalities, traditional mutual information-based methods exhibit inherent limitations in characterizing the dynamic synergistic mechanisms between cerebral regions. **Method:** This study pioneered the application of an Integrated Information Decomposition (ΦID) framework in AD brain network analysis, constructing single-sample network models based on ΦID-derived synergy metrics to systematically compare their differences with mutual information-based methods in pathological sensitivity, computational robustness, and network representation capability, while detecting brain regions with declining dynamic synergy during AD progression through intergroup *t*-tests. **Result:** The key finding are as follows: (1) synergy metrics exhibited lower intra-group coefficient of variation than mutual information metrics, indicating higher computational stability; (2) single-sample reconstruction significantly enhanced the statistical power in intergroup difference detection; (3) synergy metrics captured brain network features that are undetectable by traditional mutual information methods, with more pronounced differences between networks; (4) key node analysis demonstrated spatiotemporal degradation patterns progressing from initial dysfunction in orbitofrontal–striatal–temporoparietal pathways accompanied by multi-regional impairments during prodromal stages, through moderate-phase decline located in the right middle frontal and postcentral gyri, to advanced-stage degeneration of the right supramarginal gyrus and left inferior parietal lobule. ΦID-driven dynamic synergy network analysis provides novel information integration theory-based biomarkers for AD progression diagnosis and potentially lays the foundation for pathological understanding and subsequent targeted therapy development.

## 1. Introduction

Alzheimer’s disease (AD) is a prevalent neurodegenerative disorder characterized by progressive neurodegeneration and connectivity deterioration [1]. These pathological features impair the brain’s structural and functional networks, resulting in severe cognitive and behavioral impairments. Clinically, early-stage AD is typically characterized by memory impairment caused by medial temporal lobe neurodegeneration [2], accounting for 60–70% of dementia cases. As the disease progresses, this neurodegeneration gradually spreads to the temporal and parietal cortices, eventually affecting most of the cortex [3]. Current diagnostic methods for AD are imperfect, primarily relying on psychological tests and clinical observations. Additional diagnostic tools may aid clinicians in enhancing diagnostic accuracy and advancing pathophysiological understanding.

Functional MRI (fMRI) measures neuronal activity through blood-oxygen-level-dependent (BOLD) signal fluctuations [4,5]. Since its inception in the early 1990s, this non-invasive imaging modality has been particularly valuable for investigating neurodegenerative processes in Alzheimer’s disease [6], where it enables in vivo characterization of network-level pathology that parallels histopathological staging. Resting-state fMRI examines the brain’s state during relaxation, essentially reflecting intrinsic spontaneous brain activity [7], and its data analysis has yielded many important insights. In fMRI research, modeling the brain as a “graph” provides a powerful analytical framework. This approach parcellates the brain into nodes (regions of interest, ROIs) and constructs whole-brain networks by calculating edges (functional connectivity between regions). Graph theory-based complex brain network analysis techniques allow systematic quantification of global and local topological properties, thereby elucidating pathological mechanisms underlying neurological diseases [8]. Traditional metrics for functional connectivity (e.g., Pearson correlation coefficients) are conceptually straightforward and appealing, essentially reflecting similarity in signal fluctuations between brain regions. However, such analyses deviate from our primary objective: understanding how brain regions integrate information and specialize.

The conceptualization of the brain as a distributed information processing system has established a robust theoretical framework for analyzing neural network dynamics [9]. The common method uses mutual information (MI) to construct functional connectivity, which calculates based on probability distributions to measure the dependency between two random variables, with advantages in handling nonlinear relationships and unrestricted variable types. However, it cannot adequately describe the numerous dynamic phenomena that may emerge in complex interacting systems like the human brain. Dynamic functional connectivity surpasses traditional static approaches, demonstrating that static connectivity contains distinct patterns with different cognitive roles [10,11]. Similarly, “integrated information decomposition” (ΦID) provides unique insights into specialized information processing patterns [12].

The synergistic atoms derived from ΦID provide a unique perspective for investigating complex network reorganization in Alzheimer’s disease (AD) by quantifying the dynamic capacity of multi-regional collaborative information processing. AD pathology manifests not merely as isolated degeneration of specific brain areas, but rather through progressive disintegration of cross-regional synergistic processing capabilities. Synergy metrics grounded in information integration theory can analyze dynamic collaborative interactions between brain regions—a critical dimension obscured by conventional mutual information approaches, which is essential for capturing AD-characteristic distributed network dysfunction.

This study pioneers the application of dynamic synergistic information based on ΦID into AD’s functional connectome analysis and investigates key altered brain regions during AD progression from an information processing perspective. The results demonstrate stage-specific regional degeneration patterns, providing novel insights into Alzheimer’s disease pathogenesis.

## 2. Materials and Methods

### 2.1. Participants

The dataset was obtained from the Alzheimer’s Disease Neuroimaging Initiative (ADNI) (ADNI: https://adni.loni.usc.edu (accessed on 8 November 2023)) [13], with approval from institutional review boards and informed consent obtained from all participants. The ADNI was launched in 2003 as a public–private partnership, led by Principal Investigator Michael W. Weiner, MD. The primary goal of ADNI has been to test whether serial magnetic resonance imaging (MRI), positron emission tomography (PET), other biological markers, and clinical and neuropsychological assessment can be combined to measure the progression of mild cognitive impairment (MCI) and early Alzheimer’s disease (AD). The ADNI consortium employs rigorous exclusion criteria encompassing major psychiatric disorders, substance dependence, and neurological comorbidities (e.g., Parkinson’s disease and multiple sclerosis). All participants underwent screening for MRI contraindications and had clinically stable medication regimens for ≥4 weeks prior to scanning. For up-to-date information, see www.adni-info.org (accessed on 8 November 2023).

The data include 34 CN (cognitively normal), 60 EMCI (early mild cognitive impairment), 59 LMCI (late mild cognitive impairment), and 50 AD (Alzheimer’s disease) subjects aged 60–80 years.

### 2.2. Data Acquisition

Functional images were obtained from ADNI database with 3T field strength using Philips Medical Systems scanners (Amsterdam, The Netherlands), matrix size = 64 × 64, voxel size = 3.3 × 3.3 × 3.3 mm^3^, GR pulse sequence, 48/36 slices per subject with 3.3 mm gap, 140 volumes, TE = 30.0 ms, TR = 3001.0 ms.

Structural images were obtained from ADNI database with 3T field strength using Philips Medical Systems scanners, matrix size = 256 × 256, GR pulse sequence, slice gap = 1.2 mm, TE = 3.1 ms, TR = 6.8 ms, TI = 0.0 ms.

### 2.3. Preprocessing

Preprocessing of datasets included: (1) Reorient: Check and exclude distorted images, then reset the origin of remaining fMRI data; (2) Slice-timing correction: Apply top-down inter-leaved slice timing correction to address temporal discrepancies (default inter-leaved slice order: [1, 3, 5, …, 47/35, 2, 4, 6, …, 48/36]); (3) Head motion correction: Correct for head movements in six directions (x/y/z translation and rotation), exclude subjects with mean framewise displacement > 0.5 mm; (4) Coregister: Align T1-weighted structural images with functional images; (5) Segmentation: Segment coregistered structural images into gray matter, white matter, CSF, bone and others; (6) Normalization: Affine transformation to MNI space with 1 mm structural and 3 mm functional resampling; (7) Smoothing: Apply 6 × 6 × 6 Gaussian kernel smoothing to enhance SNR. All steps were implemented using DPABI [14] and SPM12 (v7771, http://www.fil.ion.ucl.ac.uk/spm (accessed on 4 May 2023)).

### 2.4. Mutual Information and Synergy Computation

In information theory, Shannon entropy H(X) [15] quantifies the uncertainty associated with random variable X. Higher entropy indicates greater uncertainty and information content. The entropy is defined as:

(1) $$H(X)=-\sum_{x\in \alpha_{x}}p\left(x\right)\log_{2}p\left(x\right)$$

For two random variables X and Y, mutual information $I\left(X;Y\right)$ can be defined using Shannon entropy [15], representing the information one variable contains about another. The mutual information is given by:

(2) $$I\left(X;Y\right)=H\left(X\right)+H\left(Y\right)-H\left(X,Y\right)=\sum_{x\in X}\sum_{y\in Y}{p\left(x,y\right)\log}_{2}\frac{p\left(x,y\right)}{p\left(x\right)p\left(y\right)}$$

Partial Information Decomposition (PID) extends Shannon’s theory by decomposing mutual information into unique, synergistic, and redundant components between source variables relative to a target [16]:

(3) $$I\left(S;R_{1},R_{2}\right)=red+un1+un2+syn$$

**Figure 1:**
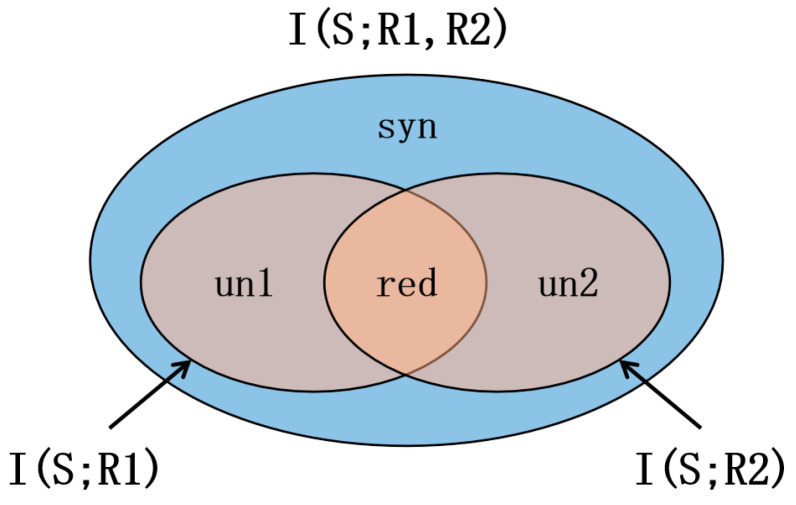
Example diagram of partial information decomposition.

A schematic representation of the PID decomposition into four distinct information components is provided in Figure 1.

Consider a system with two interdependent variables that co-evolve over time. The information between past and future states can be quantified by time-delayed mutual information (TDMI):

(4) $$TDMI=I(X_{t-\tau };X_{t})=I(X_{t-\tau }^{1},{\;X}_{t-\tau }^{2};{\;X}_{t}^{1}{,\;X}_{t}^{2})$$

**Figure 2:**
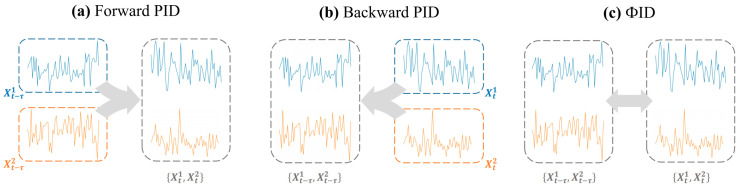
(**a**) Forward PID; (**b**) backward PID; and (**c**) integrated information decomposition.

Integrated Information Decomposition (ΦID) [12] extends PID by forward PID and backward PID (See Figure 2), decomposing TDMI into unique, synergistic, and redundant components.

Although PID provides a decomposition framework, it does not specify computation methods for components. Since linear Gaussian models adequately describe fMRI time series, we adopt MMI-PID decomposition based on previous neuroscience applications, using minimum mutual information (MMI) to compute double-redundancy [17,18]:

(5) $$Red(X_{t-\tau };X_{t})={min}_{ij}I(X_{t-\tau }^{i};X_{t}^{j})$$

For two time series, a set A can represent system states:

(6) A={un1, un2, red,syn}

**Figure 3:**
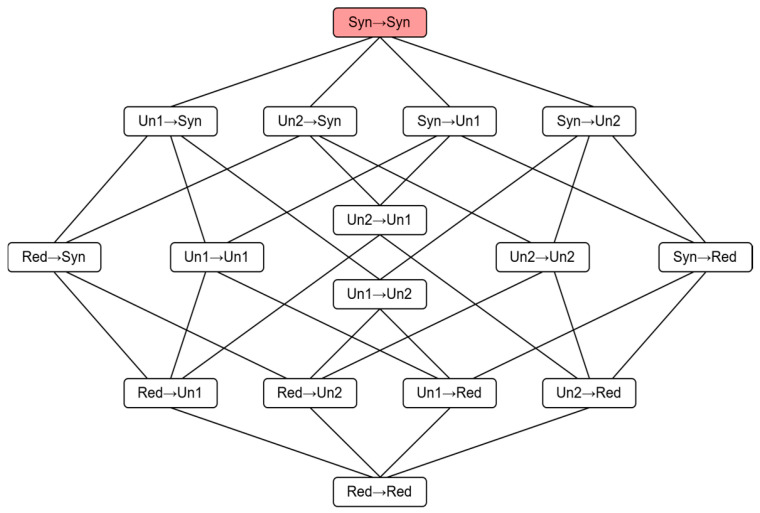
The information lattices of 16 atoms. $I_{\partial }^{\alpha \to \beta }$ denotes atomic information quantity at position $\alpha \to \beta ,{\;I}_{\cap }^{\alpha \to \beta }$ denotes the sum of information atoms at position α→β and below.

Both forward and backward PID in the system can be decomposed into four components: redundancy (red), unique1 (un1), unique2 (un2), and synergy (syn), resulting in 16 possible interaction patterns. Their arrangement follows ordered information lattices (see Figure 3). Taking α as source and β as target, $I_{\cap }^{\alpha \to \beta }$ represents the double-redundancy function indicating the sum of information atoms at position α→β and below. $I_{\partial }^{\alpha \to \beta }$ denotes atomic information quantity at position α→β. For given α→β∈A×A, it satisfies:

(7) $$I_{\cap }^{\alpha \to \beta }=\left\{\begin{array}{c} Red\left(X_{t-\tau }^{\alpha_{1}},\ldots ,X_{t-\tau }^{\alpha_{J}};X_{t}^{\beta_{1}}\right)\;\;\;if\;\;K=1,\\ Red\left(X_{t-\tau }^{\alpha_{1}};X_{t}^{\beta_{1}},\ldots ,X_{t}^{\beta_{K}}\right)\;\;\;\;if\;\;\;J=1,\\ I\left(X_{t-\tau }^{\alpha_{1}};X_{t}^{\beta_{1}}\right)\;\;\;\;\;\;\;\;\;\;\;\;\;if\;\;\;J=K=1. \end{array}\right.$$

For the inherent ordering $\alpha^{\prime }\to \beta^{\prime }≼\alpha \to \beta$ in A×A, it satisfies:

(8) $$I_{\cap }^{\alpha \to \beta }=\sum_{\alpha^{\prime }\to \beta^{\prime }≼\alpha \to \beta }I_{\partial }^{\alpha^{\prime }\to \beta^{\prime }}\mathrm{and}\;I_{\partial }^{\alpha \to \beta }=I_{\cap }^{\alpha \to \beta }-\sum_{\alpha^{\prime }\to \beta^{\prime }≺\alpha \to \beta }I_{\partial }^{\alpha^{\prime }\to \beta^{\prime }}$$

These relationships form a 15-equation system: 6 are given by $Red\left(X_{t-\tau }^{1},X_{t-\tau }^{2};X_{t}\right)$, $Red\left(X_{t-\tau }^{1},X_{t-\tau }^{2};X_{t}^{1}\right)$, $Red\left(X_{t-\tau }^{1},X_{t-\tau }^{2};X_{t}^{2}\right)$ (with 3 backward PIDs); 9 are given by standard mutual information $I\left(X_{t-\tau }^{i};X_{t}^{j}\right),I\left(X_{t-\tau }^{1},X_{t-\tau }^{2};X_{t}^{j}\right),I\left(X_{t-\tau }^{i};X_{t}^{1},X_{t}^{2}\right)$ and $I\left(X_{t-\tau }^{1},X_{t-\tau }^{2};X_{t}^{1},X_{t}^{2}\right)$ for $i,j=\left\{1,2\right\}$. The last one is given by predefined double-redundancy function.

Finally, atomic $I_{\partial }^{syn\to syn}$ (Syn) is selected as persistent synergy component to construct functional connectivity matrix. In contrast to traditional mutual information (MI) that quantifies pairwise statistical dependencies, the atomic $I_{\partial }^{syn\to syn}$ capture the collaborative information processing capability between brain regions beyond mere pairwise interactions. This distinction can be analogized through binocular vision mechanisms: Mutual information quantifies correlations between retinal signals from individual eyes, whereas synergistic information characterizes the emergent depth perception arising from cortical integration of binocular disparities—a higher-order percept unattainable through simple summation of monocular or pairwise signals. The synergy metric (Syn) fundamentally quantifies the capacity for cross-regional collaborative information integration. From a neurobiological perspective, elevated synergy indicates that interacting brain regions achieve joint information processing efficacy surpassing their independent contributions [19]

For fMRI time series, synergy specifically represents the information that emerges exclusively when multiple regions jointly predict the future activity of these regions. This corresponds to the network-level pathology in Alzheimer’s disease (AD), where neurodegeneration disrupts coordinated interactions across multiple regions rather than isolated pairwise connections.

The extraction and computation of BOLD signals were performed using the Python (v3.6) library Nilearn (v0.10.2). The computation of mutual information and synergy was conducted using the information-theoretic toolbox JIDT (v1.5).

### 2.5. Computing Functional Connectivity Matrix

**Figure 4:**
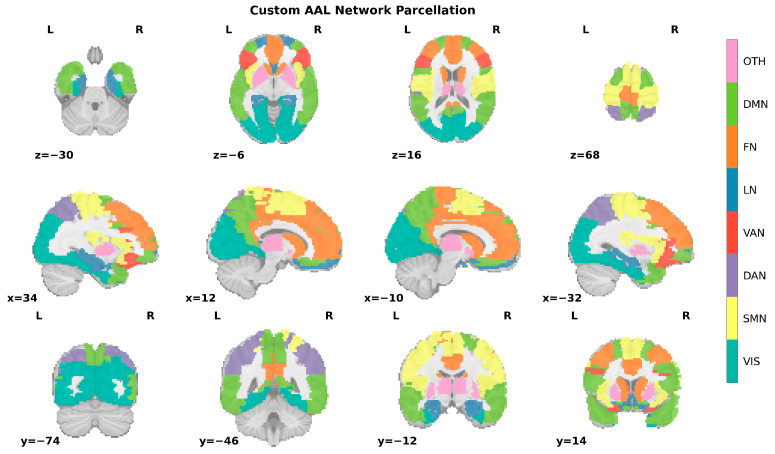
AAL parcellation mapped to Yeo’s networks. After excluding the cerebellum, 90 brain regions of the AAL template were divided into 8 brain networks according to Yeo’s 7-network scheme.

The functional connectivity matrix was computed using the Automated Anatomical Labeling (AAL) atlas proposed by Tzourio-Mazoyer et al. [20], which parcellates the brain into 116 regions. Functional brain networks represent sets of neural elements showing correlated activity, ranging from cell populations to macroscopic regions [21]. To investigate synergistic information at network level, we reorganized AAL regions according to Yeo’s 7-network scheme [22], excluding 26 cerebellar regions, resulting in eight networks: Visual network (VIS), Sensorimotor network (SMN), Dorsal attention network (DAN), Ventral attention network (VAN), Limbic network (LN), Frontoparietal network (FN), Default mode network (DMN) and Other networks (OTH) (See Figure 4). Due to limited nodes in VAN, we focused on other networks excluding OTH and VAN.

BOLD signals were averaged across voxels within each region. We extracted signals and computed pairwise mutual information and synergy to construct individual connectivity matrices by using Nilearn toolkit.

### 2.6. Single-Subject Network Reconstruction

Due to high inter-individual variability in functional connectivity matrices potentially obscuring disease-stage differences, we adopted Huang et al.’s method [23] to reconstruct matrices for group-level comparisons. First computed the average reference matrix for healthy controls:

(9) $${CN}_{avg}=\frac{1}{N}\sum_{k=1}^{N}{CN}_{k}$$

where ${CN}_{k}$ is the connectivity matrix of the k-th healthy subject, N is the number of controls.

Then, compute the differential weight matrix between subjects and *CN* average by:

(10) $$F[i][j]=\frac{\left|R[i][j]-{CN}_{avg}[i][j]\right|}{\sqrt{\frac{{\left(R[i][j]\right)}^{2}+{\left({CN}_{avg}[i][j]\right)}^{2}}{2}}},\;\mathrm{and}\;W[i][j]=1-\frac{e^{2F[i][j]}-1}{e^{2F[i][j]}+1}$$

where R is the computed connectivity matrix, R[i][j] denotes matrix element, W is the group-difference weight matrix.

Multiply the weight matrix with average matrix to obtain reconstructed connectivity matrix X:

(11) $$X[i][j]=W[i][j]\times {CN}_{avg}[i][j]$$

### 2.7. Graph Theory Metrics and Statistical Analysis

Characteristic path length: quantifies global connectivity efficiency as mean shortest path between all node pairs:

(12) $$L=\frac{1}{n}\sum_{i\in N}L_{i}=\frac{1}{n}\sum_{i\in N}\frac{\sum_{j\in N,j\neq i}d_{ij}}{n-1}$$

where distances $d_{ij}$ are computed via Dijkstra’s algorithm [24], n is the total number of nodes.

Weighted Clustering coefficient: Measures node clustering. For unweighted graphs, it is the ratio of existing edges to possible edges among neighbors [25]:

(13) $$C\left(i\right)=\frac{2e_{i}}{k_{i}\left(k_{i}-1\right)}$$

where $k_{i}$ is node degree, $e_{i}$ is existing edges between neighbors.

Weighted clustering coefficient incorporates edge weights to better reflect local clustering [26]:

(14) $$C_{w}\left(i\right)=\frac{\sum_{j,k}{\left(w_{ij}\cdot w_{jk}\cdot w_{ki}\right)}^{1/3}}{k_{i}\left(k_{i}-1\right)}$$

where $w_{ij}$ is edge weight between node *i* and *j*.

Global C is average of local coefficients:

(15) $$C=\frac{1}{n}\sum_{i=1}^{n}C_{w}\left(i\right)$$

Communication capacity: we define this metric to quantify a node’s information transfer capability:

(16) $$D\left(i\right)=\sum_{i\neq j}\frac{w_{ij}}{{dist}_{ij}}$$

where ${dist}_{ij}$ represents euclidean distance.

Degree centrality: measures node importance [27]:

(17) $$C_{D}\left(i\right)=\frac{k_{i}}{n-1}$$

In statistical analysis, sparse networks were constructed by retaining top 10% edge weights. Next, we computed the distribution of average weighted clustering coefficient and average characteristic path length at the networks level to investigate differences between MI and Syn-based methods. We calculated effect sizes using Cohen’s d with the following formula:

(18) $$d=t\times \sqrt{\frac{1}{n_{1}}+\frac{1}{n_{2}}}$$

**Figure 5:**
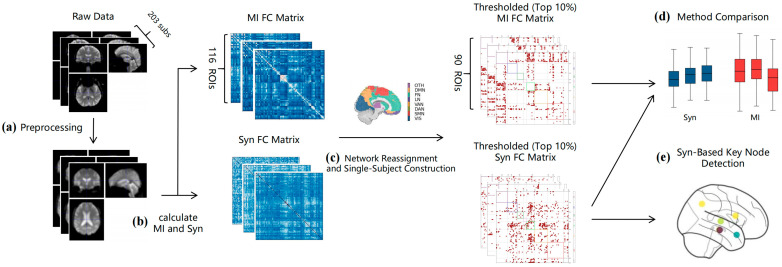
The steps followed for the analysis pipeline: (**a**) standard preprocessing for fMRI and T1-weighted MRI data (203 subjects); (**b**) construction of individual functional connectivity matrices using mutual information and synergy metrics; (**c**) parcellation of 90 AAL-defined regions into 8 resting-state networks, followed by sparse matrix construction with top 10% connection strength thresholding; (**d**) systematic comparison between MI and Syn methods across three domains: pathological sensitivity, computational robustness, and network characterization capability; (**e**) synergy-driven inter-stage analysis identifying nodes with stage-specific decline in dynamic synergy capacity during AD progression.

We performed nodal-level *t*-tests comparing communication capacity and degree centrality between groups, with false discovery rate (FDR) correction applied across all 90 cortical AAL nodes (*p* < 0.05). Among statistically significant nodes, we further selected those with larger effect sizes. Some metrics were computed using NetworkX [28]. A schematic of the applied framework is presented in Figure 5.

## 3. Results

### 3.1. Single-Sample Reconstruction Result Verification

The validation protocol involved the following: (a) thresholding reconstructed functional connectivity matrices (retaining top 10% edges); (b) computing global weighted clustering coefficients; and (c) conducting intergroup comparisons to assess improvements in absolute t-value magnitudes and *p*-value significance, with the results presented in Table 1 and Table 2.

According to the intergroup test results, the *p*-values showed noticeable decreases after single-sample reconstruction, with Syn-based intergroup tests exhibiting smaller *p*-values. This indicates that the statistical power of intergroup tests was significantly enhanced after reconstruction, and the Syn-based reconstructed functional connectivity matrices better reflected differences between disease stages. The reconstruction process resulted in significantly increased Cohen’s d effect sizes, indicating that synergy-based reconstruction enhances sensitivity to detect clinically meaningful differences between early disease stages. This improved sensitivity could enable earlier intervention by identifying EMCI patients exhibiting accelerated network degradation.

### 3.2. Inter-Method CV Stability Analysis

We compared synergy and mutual information methods by analyzing their result distributions. Using the top 10% edges from connectivity matrices, we computed CV (SD/mean) for average characteristic path length and global weighted clustering coefficient to assess dispersion. The results are presented in Table 3 and Table 4.

The CV results show that the Syn-based method produces more concentrated distributions, indicating greater stability. This enhanced stability is critical for tracking network deterioration trajectories in AD progression, serving as a prerequisite for ensuring data reliability in pathological analyses.

### 3.3. Resting-State Network Metric Comparison

**Figure 6:**
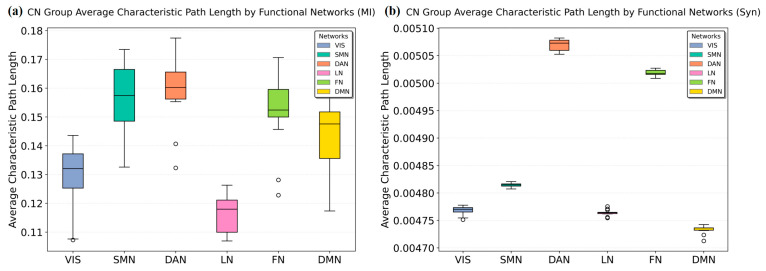
Average characteristic path length between resting-state networks and other brain regions: (**a**) average characteristic path length between individual resting-state networks and other brain regions computed using MI; (**b**) average characteristic path length between individual resting-state networks and other brain regions computed using Syn. The circles in the box plots represent outliers.

**Figure 7:**
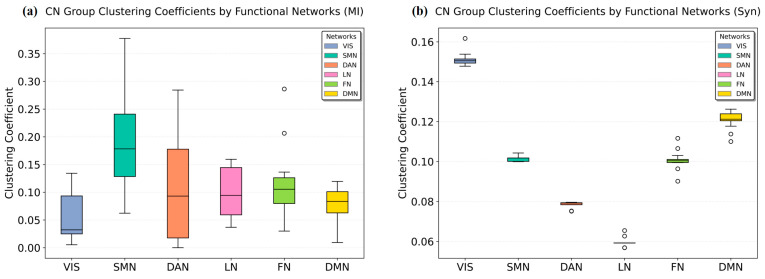
Weighted clustering coefficient distributions across functional networks: (**a**) average clustering coefficients of individual resting-state networks computed using MI; (**b**) average clustering coefficients of individual resting-state networks computed using Syn. The circles in the box plots represent outliers.

We compared MI and Syn-based methods at the network level using average path length and weighted clustering coefficients. The results from Figure 6 and Figure 7 demonstrate that the Syn-based method reveals more pronounced inter-network differences across different metrics.

Through the calculation results of Figure 6, it can be seen that the Syn and MI calculations exhibit distinct distribution patterns. Both the MI and the Syn calculations jointly indicate relatively strong connectivity between VIS/LN and other brain network regions. The Syn computation results not only show tight connections between VIS/LN and other regions but also reveal stronger connectivity between DMN/SMN and other brain areas compared with the traditional MI method.

Figure 7 shows that VIS, SMN, FN and DMN exhibit higher average weighted clustering coefficients, indicating their potential roles as core communities or information hubs, which aligns with their functional significance in the human brain. The Syn-based computations particularly highlight that the synergistic clustering coefficients of VIS, FN and DMN networks are significantly higher than those of other networks, indicating that the synergy method can reveal additional information.

### 3.4. Identifying Key Nodes in Alzheimer’s Disease Progression Using Synergy Detection

Our investigation specifically focuses on the sensitivity of Syn-based metrics in tracking neurodegenerative reorganization, employing network analysis to identify critical transition nodes across preclinical-to-dementia stages. In this section, we applied *t*-tests to pairwise compare the group differences between CN–EMCI, EMCI–LMCI, and LMCI–AD. Change Score (CS) was defined to quantify nodal capacity alterations, computed as:

(19) $$CS(k)=\frac{\bar{{stage}_{a}\left(k\right)}-\bar{{stage}_{b}\left(k\right)}}{\left|\left(\sum_{i=1}^{n}\bar{{stage}_{b}\left(i\right)}-\bar{{stage}_{a}\left(i\right)}\right)/n\right|}$$

where $\bar{{stage}_{a}\left(i\right)}$ is the capacity index of the i-th node in group a, n is the number of nodes. This parameter reflects the degree of change in a specific node relative to the overall change. Taking the CN vs. EMCI group comparison as an example, higher CS(k) values reflect greater degenerative severity at the k-th node during this transitional phase.

**Figure 8:**
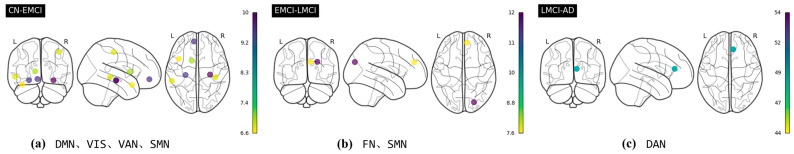
Nodes with *p* < 0.05 and CM > 6 (Communication Capacity): (**a**) CN–EMCI stage: Nodes exhibiting significant decline in dynamic synergy communication capacity localized in DMN, VIS, VAN, and SMN; (**b**) EMCI–LMCI stage: Progressive deterioration of synergy communication capacity predominantly observed in regions located in FN and SMN; (**c**) LMCI–AD stage: Advanced-stage degradation of synergy communication capacity located in DAN.

**Figure 9:**
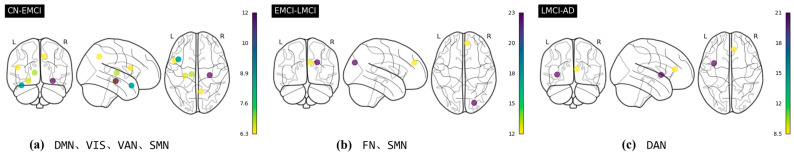
Nodes with *p* < 0.05 and CM > 6 (Degree Centrality): (**a**) CN–EMCI stage: Nodes exhibiting significant decline in synergy centrality localized in DMN, VIS, VAN, and SMN; (**b**) EMCI–LMCI stage: Progressive deterioration of synergy centrality predominantly observed in regions located in FPN and SMN; (**c**) LMCI–AD stage: Advanced-stage degradation of synergy centrality located in DAN.

Specific metrics include communication capacity and degree centrality to measure changes in information transmission capability and hub role of nodes. Finally, brain nodes with FDR-corrected *p* < 0.05 were selected, and then further refined by retaining only those with positive change scores exceeding the threshold (CS > 6). Figure 8 and Figure 9 demonstrate the spatial distribution of cerebral nodes with statistically significant metric reductions through Glass Brain visualizations, while Table 5 and Table 6 specify the anatomical nomenclature of affected regions and corresponding magnitudes of decrease.

According to Table 5 and Table 6, intergroup comparisons across disease stages revealed distinct nodes with significant degeneration. The CN–EMCI comparison identified as follows: (a) significant communication capacity reductions in seven regions (Heschl_L, Frontal_Inf_Orb_L, Rectus_L, Caudate_R, Putamen_L, Temporal_Inf_L); and (b) decreased degree centrality in six regions (Frontal_Inf_Orb_R, Temporal_Inf_L, Temporal_Pole_Sup_L, Frontal_Inf_Orb_L, Occipital_Inf_L, Frontal_Mid_Orb_R), revealing early-stage alterations in higher-order brain networks. EMCI–LMCI comparisons showed significant decreases in both communication capacity and degree centrality for Frontal_Mid_R (right middle frontal gyrus) and Postcentral_R (right postcentral gyrus). The LMCI–AD stage exhibited the following: (a) impaired communication capacity and degree centrality in SupraMarginal_R (right supramarginal gyrus); and (b) decreased degree centrality in Parietal_Inf_L (left inferior parietal lobule), highlighting late-stage network degradation. Figure 8 and Figure 9 show the localization of functionally degraded brain regions in the brain.

## 4. Discussion

This study introduces the information-theoretic concept of “Integrated Dynamic Information Decomposition” into AD fMRI analysis, reconstructing individual brain networks based on “single-subject networks”. We compared the computational distribution differences between decomposed dynamic synergy information and the traditional mutual information method, as well as their differences at the resting-state network level. Key changing nodes across disease stages were detected using decomposed dynamic synergy information, providing theoretical references for AD progression analysis and targeted therapy.

As shown in Table 1 and Table 2, the single-sample reconstruction method significantly improved statistical power, with the Syn-based between-group tests revealing more pronounced differences. This improved sensitivity could enable earlier intervention by identifying EMCI patients exhibiting accelerated network degradation. Additionally, the CA analysis results in Table 3 and Table 4 demonstrated more concentrated value distributions from the Syn-based method, indicating its computational results are more stable compared to traditional mutual information. This enhanced stability is critical for tracking network deterioration trajectories in AD progression, serving as a prerequisite for ensuring data reliability in pathological analyses.

At the resting-state network level, the Syn-based results showed different distributions and more significant network capacity differences compared to MI (see Table 7). Theoretically, brain functions manifest in inter-regional connectivity and intra-network collaboration. Syn computations can explain functional roles played by different networks. For example, VIS is a highly complex region designed to process optical information received by both eyes and convert it into recognizable informational objects. The processing of sensory inputs does not conclude within the visual system but transmits information flows through multiple pathways to the parietal cortex and temporal lobe regions for further visual stimulus processing, generating motion perception and spatial awareness [29]. This process involves a collaborative division of labor among multiple brain regions to generate abstract information related to consciousness and cognition [30]. Additionally, Bullmore et al. [31] reported that the visual network exhibits small-world properties with high clustering coefficients and short characteristic path lengths, corresponding to its efficient information integration capability, which aligns with VIS’s low average characteristic path length and high clustering coefficient in synergy measurements.

As a key player in memory, emotion, behavior, and sensory control, LN regulates many core brain functions and maintains important collaborative relationships with other brain networks including the sensorimotor network, olfactory system, gustatory system, and default mode network. Dysfunction of this system typically manifests as enhancement or reduction of core brain activities [32,33,34], while its structural lesions often lead to Alzheimer’s disease [35]. The Syn-based short-path characteristics between LN and other brain regions indicate its collaborative relationships with numerous brain regions for regulating the brain’s core functions.

The DMN was first proposed by Raichle et al. [36] and is the most well-known brain network in resting-state fMRI research, responsible for higher cognitive functions such as contemplation and introspection [37]. The DMN not only integrates information between brain regions internally [38] but also acts as a “brain hub” to some extent, constructing internal mental models by dynamically interacting with other networks to integrate cross-modal information [39]. The low average characteristic path length and high clustering coefficient features of DMN in synergy measurements align with its functions. A decline in its synergistic capacity may lead to the deterioration of abstract cognitive abilities.

The SMN is the core network responsible for integrating somatosensory inputs and motor outputs in the brain, with functions spanning multiple levels from basic motor execution to complex sensorimotor coordination [40]. As the brain’s sensory hub, SMN integrates somatosensory and visual information with VIS by connecting pathways to support spatial orientation and movement coordination [41]. It also demonstrates significant interactions with LN in the regulation of pain perception and emotion-driven motor behaviors [42], while collaborating with DAN for spatial attention orientation to execute visually guided complex actions like musical instrument performance [43]. The connections between these systems collectively form sensory cognition that guides behavior. These connectivity patterns demonstrate that SMN enables real-time sensorimotor coordination via local dense connections combined with global rapid communication, as reflected by its low characteristic path length and high synergistic clustering coefficient.

The FN can be divided into anterior and posterior regions responsible for abstract and concrete cognitive control, respectively, with intermediate regions supporting collaboration between the front and back parts by integrating control signals at different levels [44], thereby forming a more unified system. Studies also show that core FN regions (e.g., dorsolateral prefrontal cortex, inferior parietal lobule) exhibit high local connection density [45], whose high clustering coefficient in synergy measurements could explain the efficiency of dynamic collaboration between internal subnetworks.

According to Table 5 and Table 6, intergroup comparisons across disease stages revealed distinct nodes with significant alterations. The CN–EMCI comparison showed significant changes in synergy metrics across multiple brain regions. The inferior orbital frontal gyrus (Frontal_Inf_Orb) and right middle orbital frontal gyrus (Frontal_Mid_Orb_R) located in OFC participate in decision-making, reward evaluation, and emotion regulation, whose connections with amygdala and striatum are crucial for stimulus-reward association [46]. The left gyrus rectus (Rectus_L) also located in OFC collaborates with the adjacent orbitofrontal cortex and other networks (e.g., limbic system) to evaluate reward/punishment values of environmental stimuli by integrating sensory inputs and emotional signals, influencing decision-making [47]. OFC regions show significant Aβ deposition in early AD, which disrupts prefrontal-limbic system functional integration, causing default mode network (DMM) connectivity decline [48]. These findings align with significant synergy decline in Frontal_Inf_Orb_R, Frontal_Inf_Orb_L, Rectus_L, and Frontal_Mid_Orb_R during early cognitive impairment.

The left transverse temporal gyrus (Heschl_L) in the primary auditory cortex processes sound signals and decodes speech prosody, relating to semantic memory integration [49]. Its volume reduction decreases sensitivity to speech temporal resolution (e.g., phoneme discrimination), leading to prosody decoding deficits [50]. Previous studies found abnormal vowel formant bandwidths (e.g., F2, F3) in AD patients, particularly significant differences in F2/F3 parameters for vowels/a/and/u/, causing vowel articulation fuzziness and reduced speech clarity [51]. Decreased synergy communication capacity in Heschl_L can indicate an early decline in articulatory coordination during cognitive impairment.

The caudate nucleus, as a core structure of the basal ganglia, regulates motor coordination and procedural learning, participating in the reward–motivation circuit [52,53]. Ashwin et al. demonstrated that the gray matter volume of the caudate nucleus shows a significant reduction (−25%) during the mild cognitive impairment stage, which is associated with decreased expression of synaptic vesicle glycoprotein 2A (SV2A), potentially leading to impaired procedural learning and motor coordination deficits [54]. Concurrently, Guan et al. [55] reported significantly higher iron deposition in the caudate nucleus of Alzheimer’s disease (AD) patients compared to healthy elderly individuals (increased magnetic susceptibility values, *p* < 0.01), with greater spatial heterogeneity. Iron deposition showed a negative correlation with cognitive scores (MMSE), and iron metabolism abnormalities may exacerbate executive dysfunction by disrupting basal ganglia–cortical circuits.

The putamen, integrated with cortico–basal ganglia–thalamic circuits, serves as a critical hub for motor planning and habit formation [56]. Recent stereotactic electroencephalography studies indicate that the putamen plays crucial roles in higher cognitive tasks such as numerical symbol recognition and mathematical concept comprehension. For instance, significant activation in the left putamen was observed during the processing of numerical symbols (e.g., “3”) or abstract mathematical concepts [57]. Correspondingly, Guan et al. [55] also reported marked and highly specific iron deposition in the putamen of AD patients. The diminished communication capacity in both caudate and putamen during early cognitive impairment stages reveals their significance as key neural targets for AD’s multifaceted symptomatology.

The inferior temporal gyrus (Temporal_Inf), as the terminal region of the ventral visual pathway, is involved in high-level visual integration and semantic memory storage, participating in object recognition and concept formation [58,59]. Its functional abnormalities may lead to conceptual confusion in semantic dementia [60]. Synergy calculations revealed significant decreases in communication capacity and degree centrality of the Temporal_Inf, indicating its functional abnormalities in early cognitive impairment.

The left temporal pole superior temporal gyrus (Temporal_Pole_Sup_L) located in the anterior temporal lobe participates in semantic memory storage, visual cognition, and complex object recognition, integrating visual and auditory information with long-term memory to support language comprehension and concept formation [59]. In amnestic mild cognitive impairment (aMCI) patients, the amplitude of low-frequency fluctuation (ALFF) values in the left temporal pole superior temporal gyrus showed a significant reduction, correlating with episodic memory decline [61]. The left inferior occipital gyrus (Occipital_Inf_L), belonging to the visual association cortex, participates in high-order visual processing including object recognition, spatial orientation, visuo-semantic transformation (e.g., converting text to meaning), and visual memory encoding. Single-photon emission computed tomography (SPECT) studies demonstrated reduced blood perfusion in the occipital regions of MCI patients, associated with declining visual processing functions [62]. The significant decrease in the degree centrality of Occipital_Inf_L reflects declining visual information integration efficiency in early cognitive impairment patients.

In the EMCI–LMCI comparison, synergy metric topological analysis identified significant decreases in both communication capacity and degree centrality within two regions: the right middle frontal gyrus (Frontal_Mid_R) and right postcentral gyrus (Postcentral_R). The right middle frontal gyrus (rMFG) belongs to the prefrontal cortex, serving as the convergence node of the dorsal and ventral attention networks. As a regulatory hub, it modulates both networks to control endogenous and exogenous attention and is responsible for higher cognitive functions including working memory, planning execution, attention regulation, and complex decision-making [63]. The right postcentral gyrus (rPOG), located in the anterior parietal lobe, is the core region of the primary somatosensory cortex. It receives and integrates somatosensory information (touch, pain and temperature) from the contralateral body, participating in spatial orientation and motor coordination [64]. Zhang et al. [65] found that the atrophy rate of the rMFG positively correlated with decreased working memory test scores (e.g., digit span test). Reduced fractional anisotropy in MFG white matter tracts in cognitive impairment suggests that axonal damage may exacerbate cognitive deficits. In AD patients, significantly decreased fiber bundle probability between the rPOG and superior frontal gyrus correlated with global cognitive decline [65]. fMRI studies also showed significantly decreased amplitude of low-frequency fluctuations (ALFF) in the right MFG and POG of prodromal AD patients, indicating their abnormal functional activity [66]. The significant decline in the ability of the right middle frontal gyrus and right postcentral gyrus indicates the occurrence of cognitive impairment in the course of Alzheimer’s disease.

Group comparison between LMCI and AD revealed significant decreases in communication capacity and degree centrality of the right supramarginal gyrus (SupraMarginal_R), along with reduced degree centrality of the left inferior parietal lobule (Parietal_Inf_L). The right supramarginal gyrus (rSMG) is a key region in the right parietal lobe, involved in complex action execution, auditory feedback regulation, multimodal integration, and cognitive modulation. Its dysfunction correlates with clinical phenotypes including cognitive impairment, language deficits, and spatial neglect [67]. The rSMG shows abnormal functional activity in the early AD stages. Resting-state fMRI studies found significantly decreased ALFF values in the rSMG of preclinical AD patients [68]. Zhang et al. [65] also found that decreased synaptic density in the rSMG of AD patients directly correlates with disrupted functional connectivity in the dorsal attention network (e.g., with superior frontal gyrus), leading to working memory and verbal fluency decline.

The left inferior parietal lobule (lIPL) is a core region for language, mathematics, complex action planning, and sensory integration. Its dysfunction causes language comprehension deficits, apraxia, and logical reasoning decline [69,70]. Recent studies [71] show that lIPL subregions have greater atrophy than the right side, best distinguishing AD from CN (AUC = 0.688). Its volume significantly decreases in early AD stages (e.g., LMCI) and correlates with cognitive scores. The decreased synergy capacity in this region suggests its potential as an AD biomarker.

In summary, Syn-based nodal detection reveals stage-specific alterations in synergistic functional dynamics across brain regions during Alzheimer’s disease (AD) progression. These findings demonstrate multiscale convergence with established pathological mechanisms: Amyloid-β (Aβ) plaque deposition-induced synaptic impairments disrupt nonlinear coupling among neuronal populations, while tau propagation pathways along functional connections [3] spatially overlap with the degradation patterns observed in fronto–temporo–parietal association cortices, suggesting pathological protein spreading may preferentially disrupt inter-regional collaborative capacity. The systemic decline in synergistic capability fundamentally reflects the deteriorated integration of distributed neural information. When cross-regional interactions fail to generate supra-additive (‘1 + 1 > 2’) synergistic effects, cognitive processing degenerates from high-order abstract cognition to low-order concrete responses, which establishes a novel mechanistic framework for interpreting AD-characteristic cognitive deterioration trajectories.

However, potential age-related and sex-related confounders in region-specific synergistic interactions (both intra- and inter-regional) were not systematically investigated. Additionally, information decomposition remains an active research field, where future methodological advancements may yield more significant results in neurodegenerative diseases like Alzheimer’s. Finally, as an exploratory implementation of information integration theory within the analytical framework, this study primarily serves to generate testable hypotheses for future investigations. Although the altered patterns in key brain regions identified here exhibit biologically plausible correspondence with prior histopathological studies, the quantitative associations between synergy metrics and cognitive scores (e.g., MMSE), as well as the spatial correspondence with characteristic Alzheimer’s disease (AD) pathological deposits, require further verification through multimodal studies. Future investigations will focus on the following: (1) establishing systematic mappings between synergy parameters and multi-omics biomarkers; (2) exploring the clinical utility of this framework for individualized prognostic prediction; and (3) elucidating the evolutionary trajectories of dynamic synergistic networks through longitudinal cohort studies.

## Figures and Tables

**Table 1:** Comparison of intergroup test results before and after reconstruction (MI).

		CN–EMCI	EMCI–LMCI	LMCI–AD
Before Construction	|t|	1.4382	0.2934	0.5274
	*p*	0.1739	0.7701	0.5998
	Cohen’s d	0.31	0.05	0.10
After Construction	|t|	1.4234	1.6098	1.1095
	*p*	0.1590	0.1119	0.2716
	Cohen’s d	0.30	0.30	0.21

**Table 2:** Comparison of intergroup test results before and after reconstruction (Syn).

		CN–EMCI	EMCI–LMCI	LMCI–AD
Before Construction	|t|	1.2427	1.8291	1.8453
	*p*	0.2181	0.0716	0.0699
	Cohen’s d	0.27	0.34	0.35
After Construction	|t|	2.2802	2.2554	2.7684
	*p*	0.0256	0.0272	0.0089
	Cohen’s d	0.49	0.41	0.53

**Table 3:** CV of average characteristic path length.

	Method	CN	EMCI	LMCI	AD
CV	Syn	0.0005	0.0020	0.0032	0.0028
	MI	0.0707	0.0835	0.1583	0.1712

**Table 4:** CV of global weighted clustering coefficient.

	Method	CN	EMCI	LMCI	AD
CV	Syn	0.0157	0.0182	0.0242	0.0268
	MI	0.4233	0.3223	0.3434	0.4162

**Table 5:** Key node changes during disease progression (communication capacity).

	CN–EMCI	EMCI–LMCI	LMCI–AD
Regions and CS value	Frontal_Inf_Orb_R: 10.02 Heschl_L: 9.56 Frontal_Inf_Orb_L: 9.47 Rectus_L: 6.96 Caudate_R: 6.76 Putamen_L: 6.75 Temporal_Inf_L: 6.57	Frontal_Mid_R: 12.47 Postcentral_R: 7.64	SupraMarginal_R: 49.37

**Table 6:** Key node changes during disease progression (degree centrality).

	CN–EMCI	EMCI–LMCI	LMCI–AD
Regions and CS value	Frontal_Inf_Orb_R: 11.6 Temporal_Inf_L: 8.9 Temporal_Pole_Sup_L: 6.86 Frontal_Inf_Orb_L: 6.57 Occipital_Inf_L: 6.32 Frontal_Mid_Orb_R: 6.29	Frontal_Mid_R: 23.21 Postcentral_R: 12.2	Parietal_Inf_L: 21.26 SupraMarginal_R: 8.47

**Table 7:** Comparison of resting-state network metrics between synergy (Syn) and mutual information (MI) approaches.

	Key Findings
Syn vs. MI Distribution	Syn-based analysis revealed more pronounced inter-network differences in resting-state connectivity patterns.
Characteristic Path Length	Syn methodology revealed stronger connectivity between DMN/SMN and other brain regions.
Synergistic Clustering	VIS, FN, and DMN exhibited significantly higher synergistic clustering coefficients than other networks (detectable only via the Syn method).

## Data Availability

Data used in preparation of this article were obtained from the ADNI database (adni.loni.usc.edu). The authors’ data are available upon reasonable request and with ADNI’s approval.
